# Multiscale Progressive Failure Analysis of 3D Woven Composites

**DOI:** 10.3390/polym14204340

**Published:** 2022-10-15

**Authors:** Trenton M. Ricks, Evan J. Pineda, Brett A. Bednarcyk, Linda S. McCorkle, Sandi G. Miller, Pappu L. N. Murthy, Kenneth N. Segal

**Affiliations:** 1 NASA Glenn Research Center, Cleveland, OH 44135, USA; 2 Universities Space Research Association, Washington, DC 20024, USA; 3 NASA Goddard Space Flight Center, Greenbelt, MD 20771, USA

**Keywords:** 3D woven composites, micromechanics, progressive failure, method of cells, homogenization, multiscale modeling, X-ray CT

## Abstract

Application of three-dimensional (3D) woven composites is growing as an alternative to the use of ply-based composite materials. However, the design, analysis, modeling, and optimization of these materials is more challenging due to their complex and inherently multiscale geometries. Herein, a multiscale modeling procedure, based on efficient, semi-analytical micromechanical theories rather than the traditional finite element approach, is presented and applied to a 3D woven carbon–epoxy composite. A crack-band progressive damage model was employed for the matrix constituent to capture the globally observed nonlinear response. Realistic microstructural dimensions and tow-fiber volume fractions were determined from detailed X-ray computed tomography (CT) and scanning electron microscopy data. Pre-existing binder-tow disbonds and weft-tow waviness, observed in X-ray CT scans of the composite, were also included in the model. The results were compared with experimental data for the in-plane tensile and shear behavior of the composite. The tensile predictions exhibited good correlations with the test data. While the model was able to capture the less brittle nature of the in-plane shear response, quantitative measures were underpredicted to some degree.

## 1. Introduction

Three-dimensional (3D) woven composites have emerged as attractive alternatives to traditional laminated composites by offering improved damage tolerance, customizability, and near net-shape manufacturing. This enabling technology allows structures with thick sections and/or complex geometries to be fabricated using a single preform that can then be infused with resin. Woven composites with new 3D architectures have predominantly been evaluated experimentally due to the significant challenges associated with modeling 3D woven geometries. Macromechanical modeling of these materials is particularly problematic because of their strong dependence on microstructure, coupled with their more general anisotropic behavior (compared to tape and 2D woven composites). As such, micromechanics and multiscale modeling are attractive for examination of these complex materials.

Some models have been developed to investigate the effects of damage at the various length scales present in 3D woven composites [1,2,3,4,5,6,7,8]. However, when looking at the broader 3D woven-composite modeling literature, the trend is to use highly-refined finite element models of the woven architecture at a single scale [9,10,11,12,13,14,15,16,17,18,19,20,21,22,23,24], and multiscale models are generally not employed due to prohibitive computational costs. As a result of the high computational demand for high-fidelity finite element models, particularly in the context of progressive failure analysis, it is currently challenging to perform sensitivity or optimization analyses for 3D woven composites based on this approach. Additionally, surrogate modeling may not be a reasonable solution, particularly when damage and failure are considered, due to the significant runtimes required to obtain the large numbers of training data. Finally, multiscale models offer access to the constituent (fiber/matrix) behaviors and responses, thus enabling consideration of processing-based virtual manufacturing simulations so that undesirable and non-optimal features can be eliminated.

The current work focuses on the application and validation of a multiscale modeling strategy for progressive failure analysis of 3D woven-composite repeating unit cells (RUCs). The approach is based on Multiscale Recursive Micromechanics (MsRM) as implemented within the NASA Multiscale Analysis Tool (NASMAT) software [25]. MsRM enables independent micromechanical methods to recursively call themselves to capture the material microstructural effects at lower and lower length scales. The approach is flexible enough to allow any micromechanical theory to operate at any length scale, whether calling or being called by other theories. In this work, NASMAT’s MsRM capabilities were applied to simulate an IM7-6k/RTM6 3D woven composite comprised of multiple length scales (i.e., a weave pattern made up of tows (yarns), the microstructure within a tow consisting of fibers and matrix). Since the MsRM approach tracks the stress and strain fields at every length scale in the composite, damage and failure can be predicted based on the local constituent material fields. These effects at lower length scales are homogenized to influence the material response at the higher length scales. Both the Generalized Method of Cells (GMC) and High-Fidelity Generalized Method of Cells micromechanical theories [26] were used at the various composite length scales. Additionally, a modified crack-band damage model based upon the work of refs. [27,28,29] was used to model the matrix both within and between the tows.

Realistic model geometry parameters and local fiber volume fractions were determined from detailed X-ray computed tomography (CT) scans and scanning electron microscopy (SEM) images, respectively, of the 3D woven composite. Further, imperfections evident in the X-ray CT images of the as-manufactured composite were captured in the models. These imperfections included pre-existing disbond cracks adjacent to the through-thickness binder tows, along with in-plane fiber waviness of the weft-direction tows. Results from the pristine geometry and models that accounted for binder-tow disbonds and weft-tow waviness were obtained. Uniaxial stress–strain curves of the composite for applied normal and shear strain components were compared with available mechanical test data for the composite. These results demonstrated that the MsRM can be used to readily capture nonlinear progressive failure response for 3D woven-composite-material systems.

## 2. Materials and Methods

### 2.1. Materials

**Figure 1:**
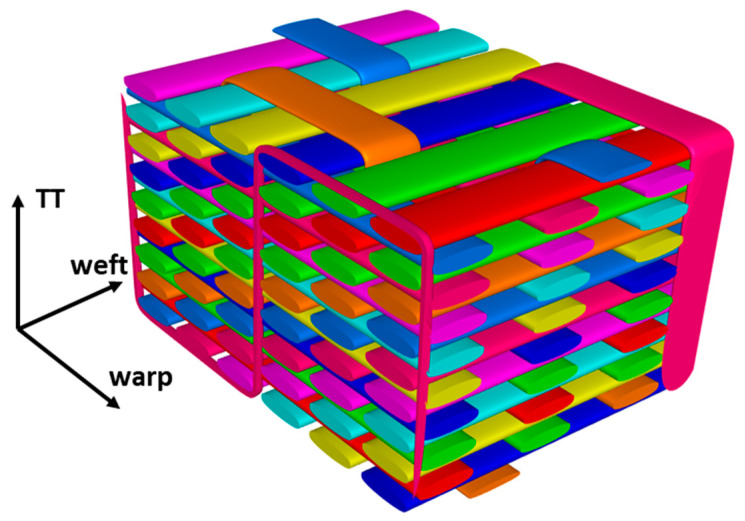
Geometry of the ideal IM7/RTM6 3D woven-composite RUC (excluding the matrix).

In this study, an IM7-6k carbon fiber/RTM6 epoxy 3D orthogonal woven composite was considered. The woven preform was manufactured by Bally Ribbon Mills (Bally, PA, U.S.A.) and was resin-infused by North Coast Composites (Cleveland, OH, U.S.A.) using a resin-transfer molding process. An RUC representing the idealized woven reinforcement geometry is shown in Figure 1. Note that the geometry is periodic in the warp and weft directions and that one of the binder tows is split across the weft-direction RUC boundary. The idealized RUC size is 8.23 mm × 4.80 mm × 3.18 mm in the warp, weft, and through-thickness (TT) directions, respectively. The RUC is comprised of 24 warp tows (8 layers, 3 columns), 54 weft tows (9 layers, 6 columns), and 3 binder tows, where each binder tow is located between adjacent columns of warp tows. Both warp and weft tows are predominantly aligned in their respective directions, while the binder tows are aligned in the warp direction at the top and bottom of the RUC but periodically traverse through the thickness of the composite.

### 2.2. Material Characterization

#### 2.2.1. Geometric Characterization Based on X-ray CT and SEM

For the IM7/RTM6 3D woven composite considered in this investigation, X-ray CT images (13.4 µm resolution) were previously generated for individual pristine specimens [30]. These data are from panel “1Z” in ref. [30], and readers are referred to that reference for more details regarding data generation and observed features. Note that the coupons whose test data are presented herein came from this same panel. In this work, the X-ray CT data were re-analyzed to identify and quantitatively measure various features of interest.

**Figure 2:**
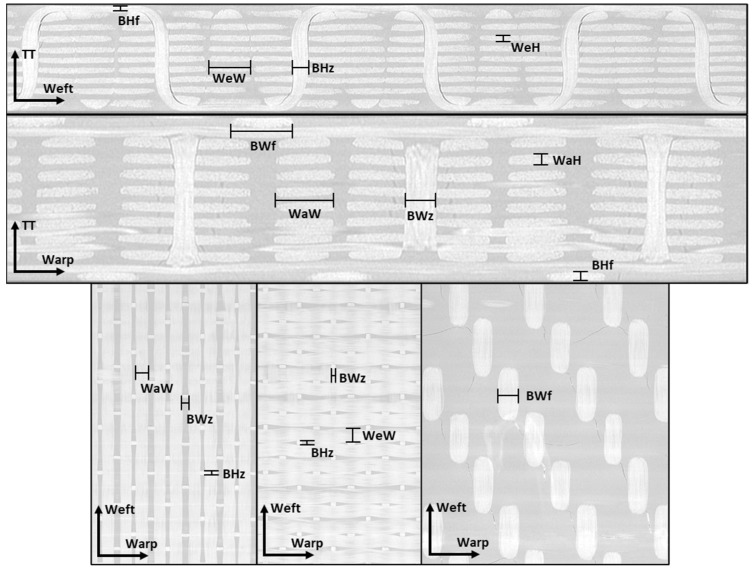
Example X-ray CT images of the IM7/RTM6 3D woven composite with dimensions measured for use in the identified multiscale RUC.

Based on features observed in the X-ray CT data, a number of key dimensions were identified and measured for use in the model RUC. Representative images are shown in Figure 2. The measured dimensions include the warp-tow width (WaW) and height (WaH) and the weft-tow width (WeW) and height (WeH). For sections of the binder tow aligned in the warp direction, the elliptical binder-tow shape is similar to the shapes of the warp- and weft-fiber tows. As the binder-tow transitions through the thickness, its width narrows and its height increases. To approximate this behavior, two sets of binder-tow dimensions were measured: the width and height in the flat warp-aligned regions (BWf and BHf, respectively) and near the mid-thickness (BWz and BHz, respectively). Statistical distributions of these eight dimensions were determined by repeatedly extracting dimensions from different locations near the appropriate tow centerline and from different cross-sectional planes if possible. For example, the warp-tow width, WaW, can be found by measuring warp tows from images normal to the warp direction or normal to the thickness direction. The mean values of the identified key dimensions were used in the construction of the 3D woven RUC for the model described in Section 2.3.3. In the future, automated techniques, such as those described in refs. [31,32,33,34,35], could be utilized to speed up the geometry-characterization process.

**Figure 3:**
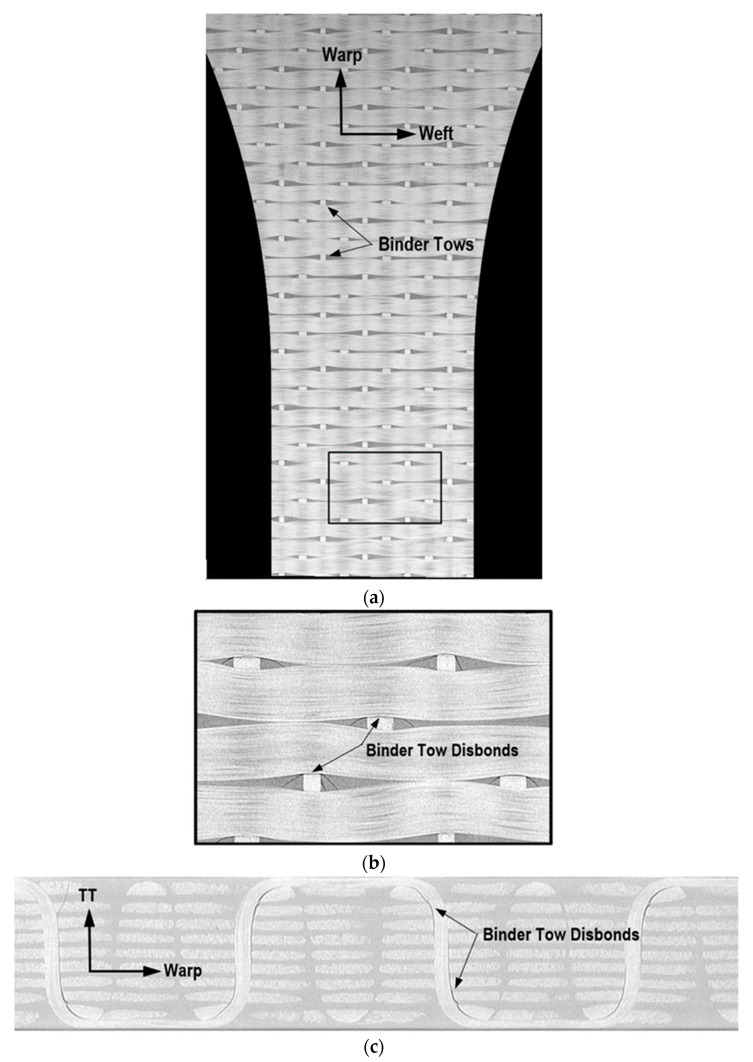
X-ray CT images showing waviness of the weft tows and disbonding of the binder tows from the surrounding matrix. (**a**) Through-thickness cross-section through the weft tows. (**b**) Detail from part (**a**). (**c**) Weft cross-section through a binder tow.

Two additional features observed in the imaging of the 3D woven composite were considered in the multiscale model: disbonding of the binder tows from the surrounding matrix and waviness in the weft tows. These are depicted in Figure 3, which shows X-ray CT images of the 3D woven composite. Figure 3a is taken from a through-thickness cross-section that passes through the weft tows, and the weft-tow waviness induced by the through-thickness binder tows is evident. Figure 3b is a detail from Figure 3a, in which the disbonding of the binder tows is shown. Figure 3c is a weft cross-section showing how these binder-tow disbonds progress through the thickness of the 3D woven composite. The disbonds appear on both sides of the binder tows in the warp direction and tend to run part way through thickness from the inner radius of the binder tow as it turns from the surface of the composite to the through-thickness direction. Note that measurements of the average fiber waviness angle were taken, but it was difficult to obtain highly accurate measurements from the X-ray CT images, as the angles were quite variable, even through the width of each weft tow. Based on these measurements, the average weft-tow waviness angle was approximated to be 1.5°, to the nearest 0.5°. Note that the warp tows were far less wavy than the weft tows. Both the weft-tow waviness and the binder-tow disbonds were included in the multiscale model of the 3D woven composite, as described in Section 2.3.3, below.

The fiber volume fraction within the 3D woven-composite tows was characterized via analysis of multiple SEM images from both the warp and weft tows. The images were segmented to separate individual fibers and matrixes using standard MATLAB-based image-processing techniques (gray-scaling and thresholding).

#### 2.2.2. Acid-Digestion Tests

Acid-digestion testing based on ASTM D3171 Procedure B [36] was used to characterize the overall fiber volume fraction and void volume fraction. A total of nine specimens were tested.

#### 2.2.3. Warp and Weft Tensile and In-Plane Shear Tests

In-plane tensile and shear tests were conducted on the woven composite at the National Institute for Aviation Research (NIAR) [37]. Uniaxial tensile tests were conducted with loading in the warp and weft directions based on ASTM Standard D3039 [38], while V-notched rail shear tests were performed according to ASTM Standard D7078 [39]. Five tensile tests (per loading direction) were performed using untabbed, 254 mm × 25.4 mm rectangular specimens. Four in-plane shear tests were performed using 55.9 mm (warp direction) × 76.2 mm (weft direction) rectangular specimens with two 90°, 12.7 mm deep notches aligned in the warp direction to give a 30.5 mm width between the notch tips. Strains were measured using a strain gauge and digital image correlation (DIC).

### 2.3. Multiscale Modeling Procedure

#### 2.3.1. Multiscale Recursive Micromechanics

**Figure 4:**
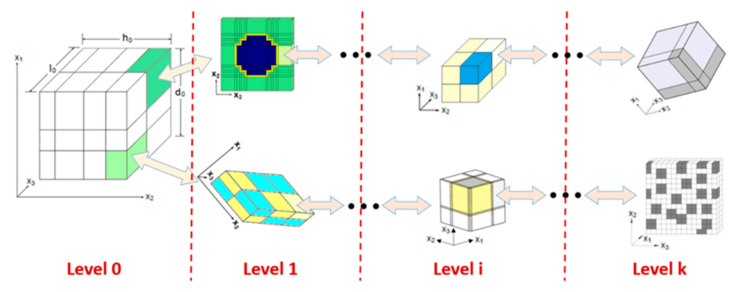
Schematic detailing of the Multiscale Recursive Micromechanics (MsRM) approach, whereby separate micromechanical models can be embedded within each other to capture structural features of interest across any number of length scales.

The MsRM approach [6,7,25] can be used to simulate materials with multiscale microstructures. Recursive procedures, subroutines, and data structures are extensively used in the NASMAT software. This permits an arbitrary number of scales to be defined, and data can be seamlessly transferred among the scales. Additionally, any micromechanical method can be utilized at any scale. Through a series of localization operations, successively lower levels are called until, ultimately, the individual constituents are reached. Once a subvolume at any level contains a constituent material, no further localizations can occur. After all localizations have occurred at a given level, properties are homogenized and passed up to the previous level. The highest level in the model, Level 0, represents the entry point for a NASMAT analysis and is where most data of interest are generated, such as the effective composite moduli and effective stress–strain response. Currently, Level 0 calculations are performed using any of the available micromechanical theories, and periodicity is assumed. To relax the assumption of global periodicity, NASMAT can be called from a third-party tool (e.g., a finite element program) where the desired boundary conditions can be applied. Each subvolume within the Level 0 RUC can call an independent Level 1 unit cell utilizing a given micromechanical theory. This process can continue up to an arbitrary *k* number of levels and is schematically shown in Figure 4.

For clarity, the mathematical details of the MsRM approach are briefly discussed. Consider any micromechanical theory utilized at a given Level, *i*. A strain concentration tensor, $A_{i}^{\left(\alpha_{i}\right)}$, can be defined that relates the local strains, $\varepsilon_{i}^{\left(\alpha_{i}\right)}$ (i.e., within a material’s subvolume), to the average (global) strains, ${\bar{\varepsilon }}_{i}$. Here, $\alpha_{i}$ denotes an individual subvolume out of the $N_{\alpha_{i}}$ total number of subvolumes. The relation between the local and average global strains is given by:

(1) $$\varepsilon_{i}^{\left(\alpha_{i}\right)}=A_{i}^{\left(\alpha_{i}\right)}{\bar{\varepsilon }}_{i}$$

The local material’s constitutive equation (assuming no inelastic or thermal effects) can be defined by:

(2) $$\sigma_{i}^{\left(\alpha_{i}\right)}=C_{i}^{\left(\alpha_{i}\right)}\varepsilon_{i}^{\left(\alpha_{i}\right)}$$

where $\sigma_{i}^{\left(\alpha_{i}\right)}$ and $C_{i}^{\left(\alpha_{i}\right)}$ are the subvolume’s stress and stiffness tensors, respectively. $\sigma_{i}^{\left(\alpha_{i}\right)}$ can be expressed in terms of ${\bar{\varepsilon }}_{i}$ by substituting Equation (1) into Equation (2):

(3) $$\sigma_{i}^{\left(\alpha_{i}\right)}=C_{i}^{\left(\alpha_{i}\right)}A_{i}^{\left(\alpha_{i}\right)}{\bar{\varepsilon }}_{i}$$

The average (global) stress tensor, ${\bar{\sigma }}_{i}$, is given by:

(4) $${\bar{\sigma }}_{i}=\sum_{\alpha_{i}=1}^{N_{\alpha_{i}}}v_{\alpha_{i}}\sigma_{i}^{\left(\alpha_{i}\right)}$$

where $v_{\alpha_{i}}$ is the volume fraction for subvolume *α_i_*. Substituting Equation (3) into Equation (4) allows ${\bar{\sigma }}_{i}$ to be expressed in terms of ${\bar{\varepsilon }}_{i}$:

(5) $${\bar{\sigma }}_{i}=\sum_{\alpha_{i}=1}^{N_{\alpha_{i}}}v_{\alpha_{i}}C_{i}^{\left(\alpha_{i}\right)}A_{i}^{\left(\alpha_{i}\right)}{\bar{\varepsilon }}_{i}$$

The effective elastic constitutive equation at Level *i* is given by:

(6) $${\bar{\sigma }}_{i}=C_{i}^{*}{\bar{\varepsilon }}_{i}$$

where $C_{i}^{*}$ is the effective stiffness tensor at Level *i*. By comparing Equations (5) and (6), $C_{i}^{*}$ is given by:

(7) $$C_{i}^{*}=\sum_{\alpha_{i}=1}^{N_{\alpha_{i}}}v_{\alpha_{i}}C_{i}^{\left(\alpha_{i}\right)}A_{i}^{\left(\alpha_{i}\right)}$$

In MsRM, the scales are linked by equilibrating the homogenized average stress, strain, and stiffness tensors at Level *i* to the local stress, strain, and stiffness tensors of a given subvolume at Level *i*−1 (with appropriate transformation to account for the potential coordinate system change from scale to scale). That is:

(8) $${\bar{\varepsilon }}_{i}=T_{2}^{i}\varepsilon_{i-1}^{\left(\alpha_{i-1}\right)},\;{\bar{\sigma }}_{i}=T_{2}^{i}\sigma_{i-1}^{\left(\alpha_{i-1}\right)},\;C_{i}^{*}=T_{4}^{i}C_{i-1}^{\left(\alpha_{i-1}\right)},\;i=1,\ldots ,k$$

where $T_{2}^{i}$ and $T_{4}^{i}$ are the appropriate second- and fourth-order coordinate transformation tensors, respectively. Hence, it is clear that starting with the lowest-scale (*k*) microstructure (see Figure 4), whose subvolumes contain only monolithic materials, the effective stiffness tensor can be calculated using any standard micromechanical theory. This stiffness tensor (after appropriate coordinate transformation) then represents the homogenized material occupying one of the subvolumes within a composite material at the next higher length scale. Given the transformed effective stiffness tensors of all subvolumes at this next higher length scale, the effective stiffness tensor of the composite at this level can be determined. This stiffness tensor can then be transformed and passed along to the next higher length scale and the process repeated until the highest length scale considered (0) is reached.

As an example, for an MsRM analysis considering three length scales (0, 1, and 2), the overall effective stiffness tensor can be written using Equations (7) and (8) as:

(9) $$C_{0}^{*}=\sum_{\alpha_{0}}v_{\alpha_{0}}{\left\{{\left(T_{4}^{1}\right)}^{-1}\sum_{\alpha_{1}}v_{\alpha_{1}}{\left[{\left(T_{4}^{2}\right)}^{-1}\sum_{\alpha_{2}}v_{\alpha_{2}}C_{2}^{\left(\alpha_{2}\right)}A_{2}^{\left(\alpha_{2}\right)}\right]}^{\left(\alpha_{1}\right)}A_{1}^{\left(\alpha_{1}\right)}\right\}}^{\left(\alpha_{0}\right)}A_{0}^{\left(\alpha_{0}\right)}$$

Superscripts on the bracketed terms are used to indicate that all variables within the brackets are functions of the subvolume indices from the next higher length scale. This includes lower scale volume fractions and subvolume indices. This notation is adopted to fully define the subvolume at a given scale as a function of its lower length-scale contributions. For example, the Level 2 effective stiffness tensor, from Equation (8), can be written as:

(10) $${\left\{{\left[C_{2}^{*}\right]}^{\left(\alpha_{1}\right)}\right\}}^{\left(\alpha_{0}\right)}={\left\{{\left[T_{4}^{2}\right]}^{\left(\alpha_{1}\right)}\right\}}^{\left(\alpha_{0}\right)}{\left\{C_{1}^{\left(\alpha_{1}\right)}\right\}}^{\left(\alpha_{0}\right)}$$

where there are distinct $C_{2}^{*}$ values for every Level 1 subvolume and distinct Level 1 composites present within each Level 0 subvolume.

In addition to multiscale homogenization, multiscale localization of the stress and strain tensors can be performed with MsRM. Multiscale localization is required to obtain local fields for handling damage (and inelasticity). For the previously described example with three length scales, the local strain tensor in an arbitrary Level 2 subvolume is expressed using Equations (1) and (8) as:

(11) $${\left\{{\left[\varepsilon_{2}^{\left(\alpha_{2}\right)}\right]}^{\left(\alpha_{1}\right)}\right\}}^{\left(\alpha_{0}\right)}={\left\{{\left[A_{2}^{\left(\alpha_{2}\right)}\right]}^{\left(\alpha_{1}\right)}\right\}}^{\left(\alpha_{0}\right)}{\left\{{\left[T_{2}^{2}\right]}^{\left(\alpha_{1}\right)}\right\}}^{\left(\alpha_{0}\right)}{\left\{A_{1}^{\left(\alpha_{1}\right)}\right\}}^{\left(\alpha_{0}\right)}{\left\{T_{2}^{1}\right\}}^{\left(\alpha_{0}\right)}A_{0}^{\left(\alpha_{0}\right)}{\bar{\varepsilon }}_{0}$$

Again, superscripts on the bracketed terms are used to indicate that all variables within the brackets are a function of the subvolume indices from the next higher length scale. This procedure can be repeated to determine the strain tensor for any subvolume at any length scale. The stress tensor can be found by simply using the strain tensor, along with the constitutive relationship given by Equation (2), at the appropriate length scale. Note that the MsRM implementation in NASMAT accounts for the influence of thermal and inelastic strains, but these additional effects are omitted from this section for simplicity. Since MsRM can seamlessly incorporate multiple length scales into a single analysis, it is ideal for the multiscale modeling of materials such as 3D woven composites that exhibit identifiable microstructures across multiple length scales.

#### 2.3.2. Crack-Band Progressive Damage Model

While fiber filament failure was modeled as sudden (brittle) at the lowest level considered (maximum stress criteria, with failure properties given in Section 2.3.3), the crack-band model allows the matrix to be progressively damaged [27]. Herein, the crack-band model was applied at the lowest, constituent level for the resin matrix material. This version of the theory assumes that the crack band is aligned normal to the local material coordinates as opposed to being aligned normal to the direction of maximum principal stress, as in ref. [28].

**Figure 5:**
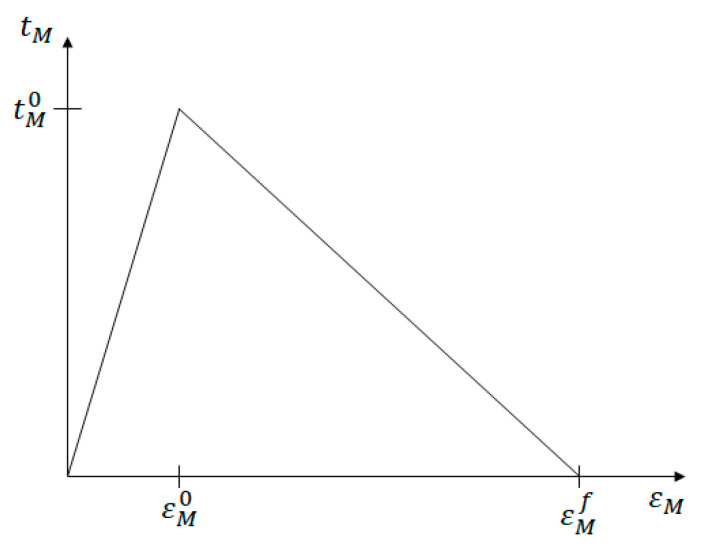
Mixed-mode traction–strain law used to govern local crack-band damage.

The mixed-mode traction–separation law for the crack-band model follows the formulation in ref. [29]. For complete details on the current implementation, the reader is referred to ref. [7]. Quadratic failure criteria are utilized to initiate a crack band within the subvolume for a monolithic matrix material ($\alpha_{m}$):

$${\left(\frac{\langle \sigma_{11}^{\left(\alpha_{m}\right)}\rangle }{X_{m}}\right)}^{2}+{\left(\frac{\tau_{12}^{\left(\alpha_{m}\right)}}{Y_{m}}\right)}^{2}+{\left(\frac{\tau_{13}^{\left(\alpha_{m}\right)}}{Y_{m}}\right)}^{2}\geq 1,$$

(12) $${\left(\frac{\langle \sigma_{22}^{\left(\alpha_{m}\right)}\rangle }{X_{m}}\right)}^{2}+{\left(\frac{\tau_{12}^{\left(\alpha_{m}\right)}}{Y_{m}}\right)}^{2}+{\left(\frac{\tau_{23}^{\left(\alpha_{m}\right)}}{Y_{m}}\right)}^{2}\geq 1,$$

$${\left(\frac{\langle \sigma_{33}^{\left(\alpha_{m}\right)}\rangle }{X_{m}}\right)}^{2}+{\left(\frac{\tau_{13}^{\left(\alpha_{m}\right)}}{Y_{m}}\right)}^{2}+{\left(\frac{\tau_{23}^{\left(\alpha_{m}\right)}}{Y_{m}}\right)}^{2}\geq 1,$$

where damage initiation is related to the normal ($X_{m}$) and shear ($Y_{m})$ matrix cohesive strengths and 〈 〉 are the Macaulay brackets. These normal and shear strengths are numerical parameters and may not necessarily be related to those obtained from experiments. The mixed-mode traction, *t_M_*, is related to an equivalent mixed-mode strain, ***ε**_M_*, through a triangular mixed-mode traction–strain law, as depicted in Figure 5. $\varepsilon_{M}^{0}$ and $t_{M}^{0}$ are the mixed-mode strain and traction, respectively, when damage initiates, and $\varepsilon_{M}^{f}$ is the mixed-mode failure strain. The orientation of the crack band can be used to determine the relationship between the tractions and the material stresses $\sigma^{\left(\alpha_{m}\right)}$ [7]. The damaged compliance matrix $S^{\left(\alpha_{m}\right)}$, where $\varepsilon^{\left(\alpha_{m}\right)}=S^{\left(\alpha_{m}\right)}\sigma^{\left(\alpha_{m}\right)}$, is expressed in terms of the scalar damage variable, *D_M_*. For instance, assuming that the crack band initiated normal to the *x*_1_ direction (as governed by the first inequality in Equation (12)), the affected components of the compliance matrix would be:

(13) $$\;\;\;\begin{array}{c} S_{11}^{\left(\alpha_{m}\right)}=\frac{\varepsilon_{11}^{\left(\alpha_{m}\right)}-\frac{\upsilon }{E}\left(\sigma_{22}^{\left(\alpha_{m}\right)}+\sigma_{33}^{\left(\alpha_{m}\right)}\right)}{ED_{M}\varepsilon_{11}^{\left(\alpha_{m}\right)}}\\ S_{55}^{\left(\alpha_{m}\right)}=S_{66}^{\left(\alpha_{m}\right)}=\frac{1}{GD_{M}} \end{array},$$

where *E* and *G* are the undamaged Young’s and shear moduli, respectively, and *ν* is the Poisson ratio for an isotropic material. If damage is initiated according to one of the other inequalities in Equation (12), then the appropriate compliance matrix, strain, and stress components must be used (Equation (13)).

Assuming the triangular mixed-mode traction–strain law, as shown in Figure 5, *D_M_* can be calculated by:

(14) $$D_{M}=\frac{\varepsilon_{M}^{0}\left(\varepsilon_{M}^{f}-\varepsilon_{M}\right)}{\varepsilon_{M}\left(\varepsilon_{M}^{f}-\varepsilon_{M}^{0}\right)}$$

The final strain energy release rate, upon complete failure, is governed by the following mixed-mode law:

(15) $$\left(\frac{G_{I}}{G_{IC}}\right)+\left(\frac{G_{II}}{G_{IIC}}\right)=1$$

where *G_I_* and *G_II_* are the mode-I and mode-II-strain energy-release rates of the material, respectively. *G_IC_* and *G_IIC_*, which are input properties, are the mode-I and mode-II fracture toughnesses of the material. Since the behavior of microcracks under mode-III conditions is still an active area of research, it is assumed in Equation (15) that the matrix has no fracture resistance under mode-III cracking similar to ref. [29]. *G_I_* and *G_II_* are calculated as:

(16) $$G_{I}=l_{c}\int^{\;}t_{I}d\varepsilon_{I}$$

(17) $$G_{II}=l_{c}\int^{\;}t_{II}d\gamma_{II}$$

where $t_{I}$ and $t_{II}$ are the mode-I and mode-II crack-band tractions, respectively, and $\varepsilon_{I}$ and $\gamma_{\mathrm{II}}$ are the mode-I (normal) and mode-II (shear) crack-band strains, respectively. *G_I_* and *G_II_* are related to the mode-specific traction–strain history and a characteristic length, *l_c_*. The total area under the mixed-mode traction–strain curve (Figure 5) is governed by Equation (15).

If the physics of the micromechanical model being employed cause the damage to localize to the size of the geometric discretization (as in finite-element analysis and HFGMC [26]), the actual discretization geometry should be used as *l_c_*. That is, the characteristic length should be set equal to the element or subcell dimension normal to the crack band. This will regulate the total energy dissipated and minimize pathological mesh-dependence [28]. The crack-band implementation within NASMAT contains an option to automatically link the characteristic length to the subcell dimensions in a consistent manner across the length scales. However, for micromechanical models that do not exhibit mesh-dependence and damage localization (such as the Mori–Tanaka method and GMC), the characteristic length may be treated as a material parameter. This latter approach has been taken herein, where the GMC micromechanical model is used at the lowest level, where the crack-band model is active. The effect of the *l_c_* parameter on the predicted 3D woven-composite behavior will be examined.

#### 2.3.3. Multiscale 3D Woven-Composite Model

**Figure 6:**
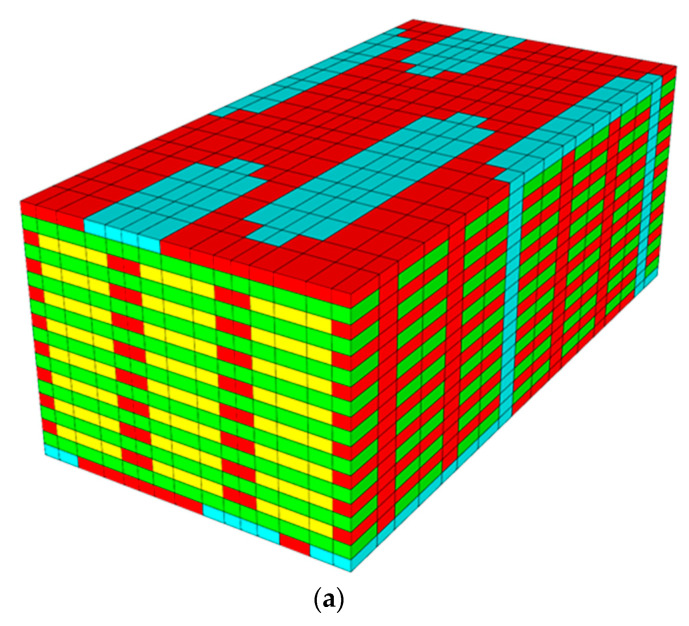

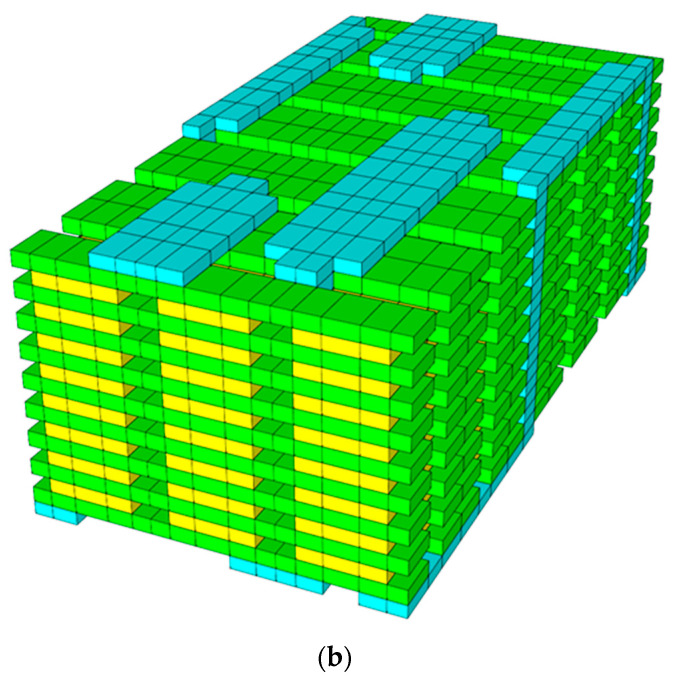
Three-dimensional woven-composite RUC showing the warp tows (yellow), weft tows (green), binder tows (blue), and pure matrix regions (red). (**a**) All materials. (**b**) Pure matrix regions removed.

The multiscale 3D woven-composite RUC is depicted in Figure 6, both with and without the pure matrix regions included. While this is a fairly coarse representation of the RUC, the key features of the woven-composite microstructural geometry have been captured. These include the ratio and sizes of the warp and weft tows and the difference between the binder-tow cross-section at the surface and as it traverses through the thickness of the composite. For reference purposes, the through-thickness (TT), weft, and warp material directions are aligned with the NASMAT global *x*_1_, *x*_2_, and *x*_3_ coordinate axes, respectively.

**Figure 7:**
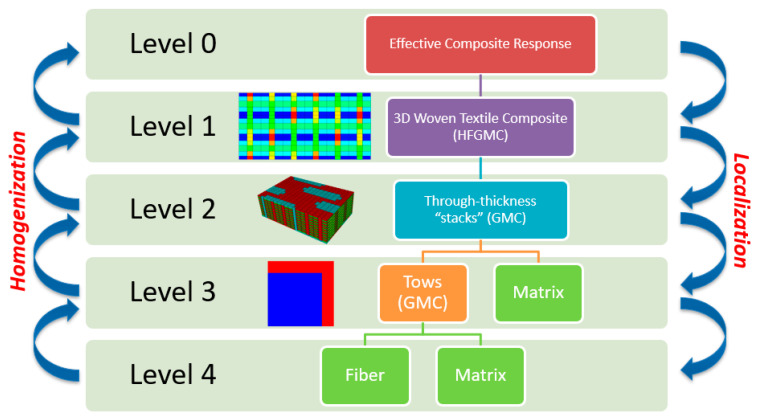
NASMAT multiscale modeling strategy for the 3D woven composite involving five levels.

Figure 7 shows schematically the full MsRM multiscale model for the 3D woven composite. Level 4 refers to the lowest length scale considered in the composite—that of the individual fibers and the matrix within the tows. These are homogenized to represent the tows using a 2 × 2 GMC RUC with a fiber volume fraction of 0.672 (as shown with a blue fiber and red matrix at Level 3 in Figure 7). At Level 3, the tows and the pure matrix regions between the tows are homogenized as through-thickness stacks, which is part of the double-homogenization procedure typically employed to compensate for the lack of shear–normal coupling in GMC when modeling woven composites [7]. Additionally, the appropriate fiber tow orientations are included in Level 3 RUCs. This results in a doubly periodic model at Level 1, where each through-thickness stack from Level 2 is depicted as a unique, anisotropic subcell. At this level, the HFGMC micromechanical model has been employed, which has been shown to give much more realistic in-plane shear predictions compared to GMC [25]. Finally, at the global scale, Level 0 represents the effective nonlinear response of the composite as the homogenized RUC behavior. Note that localization down the scales must also occur for every iteration at each increment of the applied loading, as damage occurs at the lowest levels (3 and 4) based on the local stresses, and the effect of the damage on the elastic properties is then homogenized up the scales. 

**Figure 8:**
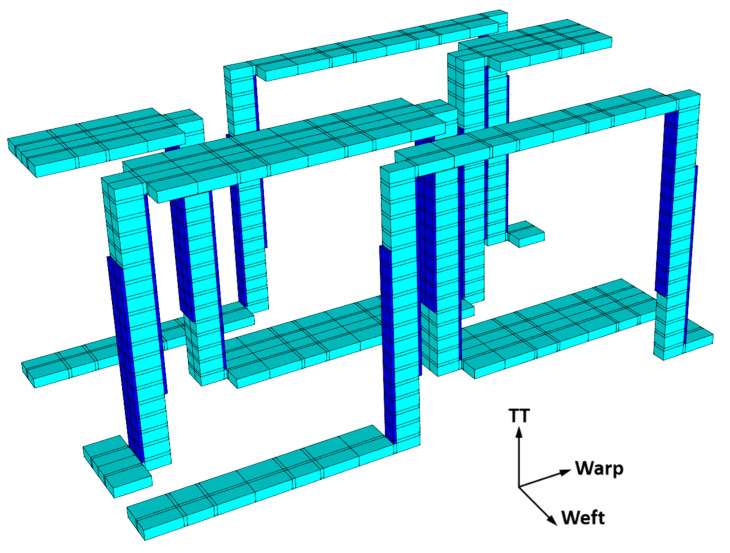
Binder tows isolated from the 3D woven-composite RUC, with dark blue indicating the locations of the subcells representing the binder-tow disbonds (see Figure 6). Note that the thicknesses of these disbond subcells have been exaggerated in this figure to make the disbonds visible.

The binder-tow disbonding discussed in Section 2.2.1 was incorporated along the binder tows, as shown in Figure 8. A number of thin subcells were added to either side of the through-thickness binder tows on the faces normal to the warp direction. This approximated the observed binder-tow disbonds shown in Figure 3. These disbond subcells are assigned a very low stiffness (matrix stiffness divided by 10^6^) for all components. Note that, because these subcells have very thin dimensions in the weft direction, they would be expected to have a minimal impact on the weft-direction response.

As an approximation of the weft-fiber waviness discussed in Section 2.2.1, all of the weft tows were assigned an in-plane angle, with alternating columns of weft tows assigned 1.5° and −1.5°. Recall that this is roughly the average weft-fiber angle measured from the X-ray CT data. This is clearly a simplification of the actual complex weft-fiber waviness shown in Figure 3. However, it should still capture the first-order effects of the waviness, as every weft tow in the model contains an imperfect angle, which is clearly reflected in the microstructural observations.

Finally, the constituent material properties employed for the RTM6 resin (treated as isotropic) and the IM7 fiber (treated as transversely isotropic) are given in Table 1. In the table, the transversely isotropic fiber properties are defined by the axial Young’s modulus, *E*_11_, the transverse Young’s modulus, *E*_22_, the axial shear modulus, *G*_12_, the axial Poisson ratio, *ν*_12_, and the transverse Poisson ratio, ν_23_. X, *Y*, and *Z* represent unique tensile, compressive, and shear strength components, respectively. Subscripts *f* and *m* are used to denote fiber and matrix properties, respectively.

## 3. Experimental Results

### 3.1. Geometic Characterization

From acid-digestion testing, the mean overall 3D woven-composite fiber volume fraction was calculated to be 0.48 (0.01 standard deviation), and the mean void volume fraction was calculated to be 0.017 (0.003 standard deviation). Note that simulations were conducted in which diffuse matrix voids were included (although not shown herein), and it was found that such a low volume fraction of voids had a negligible effect on the composite response (see ref. [6] for details about void modeling). As such, voids were not included in the models presented in this work.

**Figure 9:**
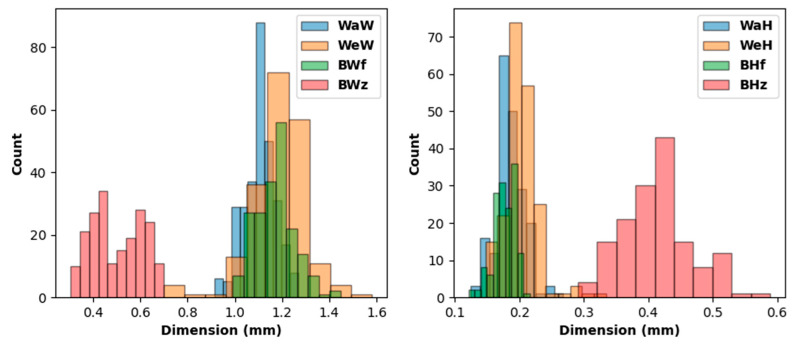
Histograms based on measurements extracted from X-ray CT images.

As mentioned in Section 2.2.1, a number of key dimensions were identified and measured from the X-ray CT data for use in the model RUC. These data are shown in Figure 9, with the means and standard deviations given in Table 2. Note that, aside from binder-tow measurements at the TT transition region, the measured tow widths and heights are similar.

**Figure 10:**
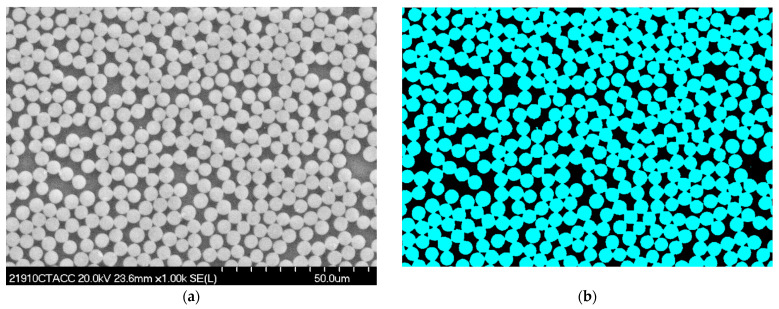
Exemplary (**a**) SEM image from inside a tow and (**b**) segmentation of the same SEM image to enable determination of the fiber volume fraction (blue—fiber; black—matrix).

**Figure 11:**
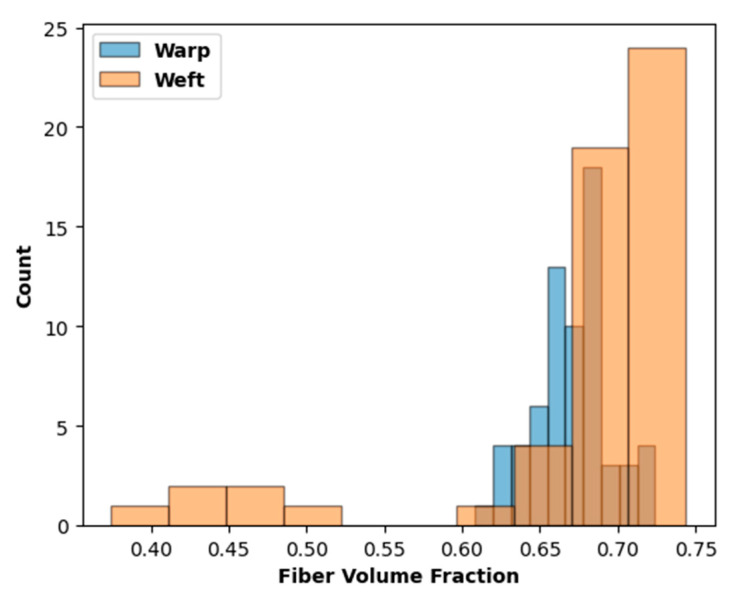
Histogram of fiber volume fractions based on segmented SEM images of the warp and weft tows within the 3D woven composite.

Figure 10a contains a representative SEM image of a fiber tow, while Figure 10b shows a segmented image. The fiber volume fraction results are given in Table 3, and a histogram of the data is shown in Figure 11. As shown, the mean fiber volume fraction values in the warp and weft tows were quite similar, so these data were combined to arrive at a mean tow-fiber volume fraction of 0.672, which was used for all tows (including the binder) in the model of the 3D woven-composite RUC.

The dimensions associated with the geometry given in Table 2 were maintained and resulted in an overall fiber volume fraction for the 3D woven composite of 0.473 (vs. a mean value of 0.48 measured with acid digestion) when using a measured fiber volume fraction of 0.672 (see Table 3) within the tows.

### 3.2. Warp and Weft Tensile and In-Plane Shear Tests

**Figure 12:**
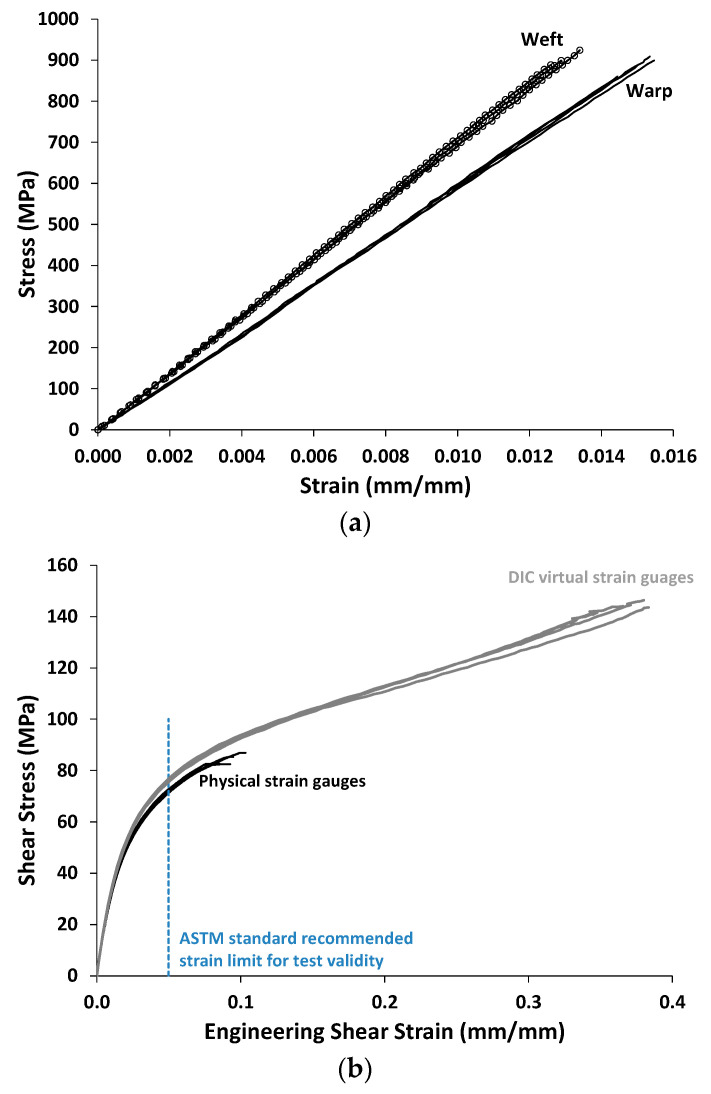
Three-dimensional woven-composite test data. (**a**) Four warp tensile tests and three weft tensile tests (DIC data only). (**b**) Four in-plane V-notched rail shear tests, with both physical strain gauge and DIC virtual strain gauge data shown.

The results from the in-plane tensile and shear tests are shown in Figure 12 and Table 4, Table 5 and Table 6. Figure 12a shows four warp and three weft tensile stress–strain curves, while Figure 12b shows three in-plane shear stress–strain curves. Some DIC data were not available near the end of the tensile tests and, as such, the plotted stress–strain curves terminated at a slightly lower stress than the measured ultimate tensile strengths (UTSs).

As expected, the weft-direction tensile modulus was greater than that for the warp direction because there are more weft tows than warp tows in the composite (and thus there are more fibers aligned with the weft direction). However, while the weft tensile modulus is approximately 20% higher than the warp (Table 4 and Table 5), the UTSs are comparable (weft average UTS = 922 MPa, warp average UTS = 902 MPa), with a difference of only 2%.

The in-plane shear stress–strain curves shown in Figure 9b indicate a much more ductile response compared to the warp and weft tensile curves. The tests progressed to very large shear strain values, although the physical strain gauges failed earlier, while the DIC strain measurements were able to continue. These shear tests also did not result in complete failure of the specimens. Furthermore, the V-notched rail shear test standard [39] indicates that these tests are only valid up to an engineering shear strain of 5%. As such, past this point, it is likely that the results shown are not reliable as material data. As indicated in Table 6, rather than shear strength, the shear stress at an engineering shear strain level of 5% is reported.

## 4. Multiscale Modeling Results and Discussion

Predictions of the nonlinear tensile and in-plane shear stress–strain responses for the 3D woven composite were made while varying the characteristic length parameter, *l_c_*. The impact of including manufacturing-induced disbonding binder tows was examined, followed by the presentation of simulations that included the observed waviness of the weft tows. Finally, the predicted responses were compared with the experimental data.

**Figure 13:**
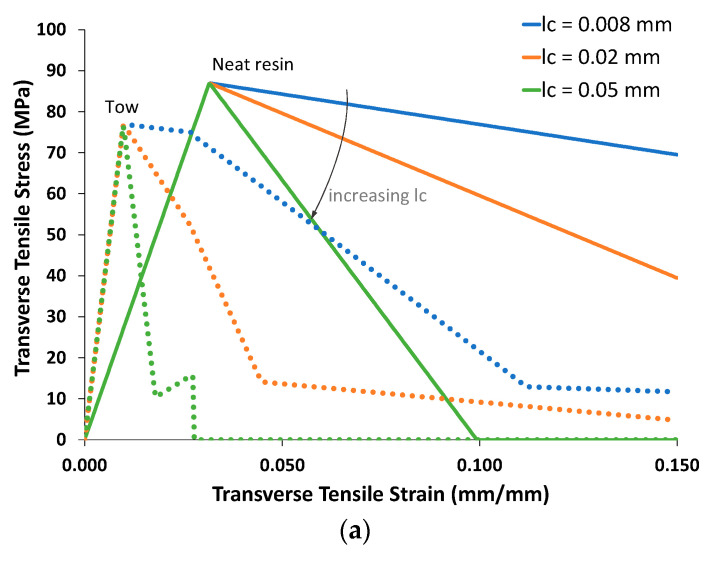

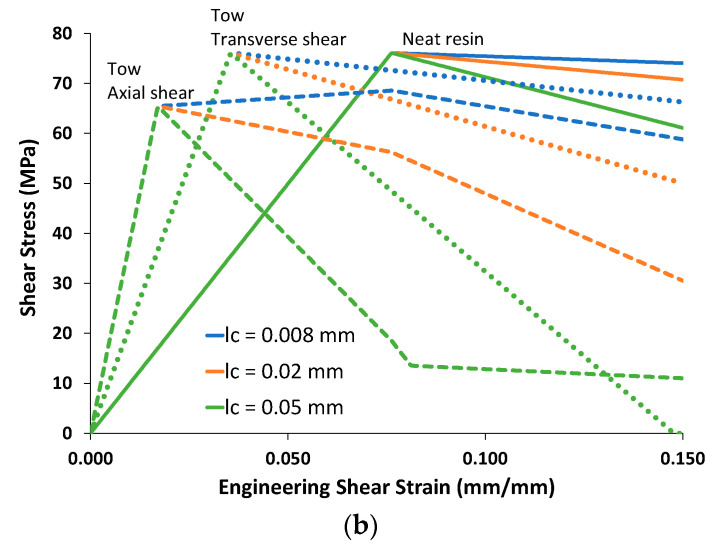
Neat resin and tow (as represented by a 2 × 2 GMC RUC, v_f_ = 0.672) responses based on the material properties given in Table 6 and different values of the characteristic length, *l_c_*. (**a**) Transverse tensile response. (**b**) Shear response.

To highlight the effects of the characteristic length parameter on the response of the materials that make up the 3D woven composite, Figure 13 shows how this parameter affects the tensile and shear responses of the RTM6 matrix material, as well as the 3D woven-composite tows, which are effectively analogous to a unidirectional composite. As discussed above, a simple 2 × 2 subcell GMC RUC, with a fiber volume fraction of 0.672, was used to model the tows.

Figure 13a, which displays the tensile response of the neat resin material and the transverse tensile response of the tow, shows that increasing *l_c_* leads to a more brittle response as the softening slope of the stress–strain response increases. The tow is modeled by an RUC that includes a brittle fiber (which, recall, is not subject to the crack-band model), and its response is thus much more brittle than that of the neat resin. The changing slopes in the tow stress–strain curves are due to different regions (subcells) of the matrix being damaged at different times.

In Figure 13b, in addition to the neat resin shear response (which is isotropic), both axial (σ_12_, with *x*_1_ as the fiber direction) and transverse (σ_23_) shear stress–strain curves are shown. As there are warp, weft, and binder tows in the 3D woven composite, the various tows will be subjected to predominantly transverse or axial shear stresses. Figure 13b shows that, again, increasing *l_c_* leads to a more brittle response, but because the applied shear loading results in mode-II-dominated simulated damage, the responses are considerably less brittle compared to the tensile loading in Figure 13a. This is because the mode-II fracture toughness is approximately four times greater than that in mode-I (see Table 1). Despite the fact that the characteristic length was varied, the mode-I and mode-II fracture toughnesses (areas under the traction–separation curves) were preserved in the crack-band model, according to the mixed-mode law in Equation (15).

It should also be noted that the responses shown in Figure 13 were generated by applying a monotonically increasing uniaxial strain. In contrast, when the resin and tow are in situ within the 3D woven-composite RUC, the loading is applied at the global scale and the local strains will be multiaxial and nonproportional during a simulation. That is, for instance, at a given point in the 3D woven composite, at a given applied global load, damage may progress significantly (e.g., local strain increasing as local stress decreases) as the model iterates. Thus, although the stress–strain curves in Figure 13 are reasonably continuous, one should not expect the global response of the 3D woven composite to be similarly free of sharp discontinuities.

### 4.1. Effect of Binder-Tow Disbonds

**Figure 14:**
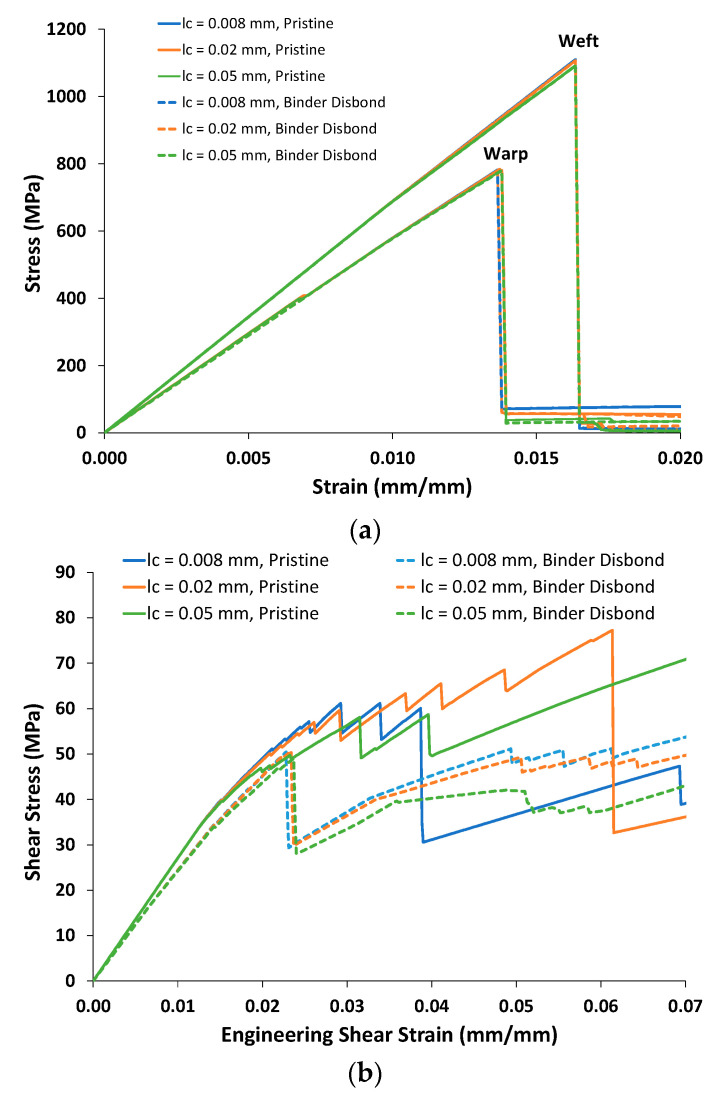
Predicted in-plane nonlinear stress–strain responses of the 3D woven composite for different values of the characteristic length, *l_c_*. (**a**) Warp and weft tension. (**b**) In-plane shear.

As described previously, pre-existing binder-tow disbonds, as shown in Figure 6, were incorporated within the multiscale model of the 3D woven composite, as shown in Figure 12, as thin subcells with very low stiffnesses. Figure 14 shows the predicted warp and weft tensile stress–strain curves, along with the predicted in-plane shear responses, for different values of the characteristic length parameter, *l_c_*, both with and without the binder-tow disbonding. From Figure 14a, it is clear that both the disbonding and the choice of *l_c_* have very minimal effects on the warp and weft tensile bahavior. This can also be seen in Table 7 and Table 8, which show the predicted orthotropic effective elastic properties of the woven composite and the predicted warp- and weft-direction ultimate tensile strengths (UTSs). *E_ij_*, *G_ij_*, and *ν_ij_* denote individual Young’s moduli, shear moduli, and Poisson ratios, respectively. Note that the effective elastic properties are independent of *l_c_*, which affects the predictions only after damage initiation (see Figure 13). As a result of the continuous tows oriented in both the warp and weft directions, the global tensile response in these directions is fairly brittle (with final failure occurring due to fiber failure). Damage does occur well before the UTS; however, its effect on the global stiffness of the composite is relatively minor.

It may be somewhat surprising that the warp direction shows such insensitivity to the binder-tow disbonding, as the disbonding is normal to the warp loading direction. However, the TT portions of the binder tows (which are disbonded) are oriented normal to the warp direction and thus do not contribute much to the composite stiffness. Furthermore, by examining the pristine warp tensile response curves in Figure 14a, it can be seen that the disbonded simulations are elastically slightly more compliant. At a stress of approximately 400 MPa (strain of 0.007), there is a slight jog in the pristine curves that slightly reduces their stiffness and causes these curves to align closely with the disbonded binder responses. This jog is caused by damage initiated in portions of the binder tows and it appears to reduce the stiffness by a similar amount to the disbonding.

In contrast to the fairly brittle predicted tensile response, as shown in Figure 14b, the 3D woven composite’s predicted in-plane shear response is very ductile. Both the choice of *l_c_* and the presence of the binder disbond have a major impact on the predicted response. This can also be seen in Table 7 and Table 8. The in-plane shear modulus, *G*_23_, is reduced by 8.1% by the binder-tow disbond, and the shear stress at 5% strain is significantly different as well. The binder disbond results in significanly lower stress after damage has been initiated, as do higher *l_c_* values, although the effect of *l_c_* is less than that of the binder-tow disbond. Note that, as was the case in the experiments (see Figure 12b), the shear predictions do not exhibit complete failure or a large stress drop. Rather, the strains become quite large as the damage continues to accumulate. Note that the shear stress at 5% strain is dependent on the damage-event sequence (i.e., the jaggedness of the stress–strain curves), and, as such, the trend in the data in Table 8 is not consistent.

### 4.2. Effect of Weft-Tow Waviness

**Figure 15:**
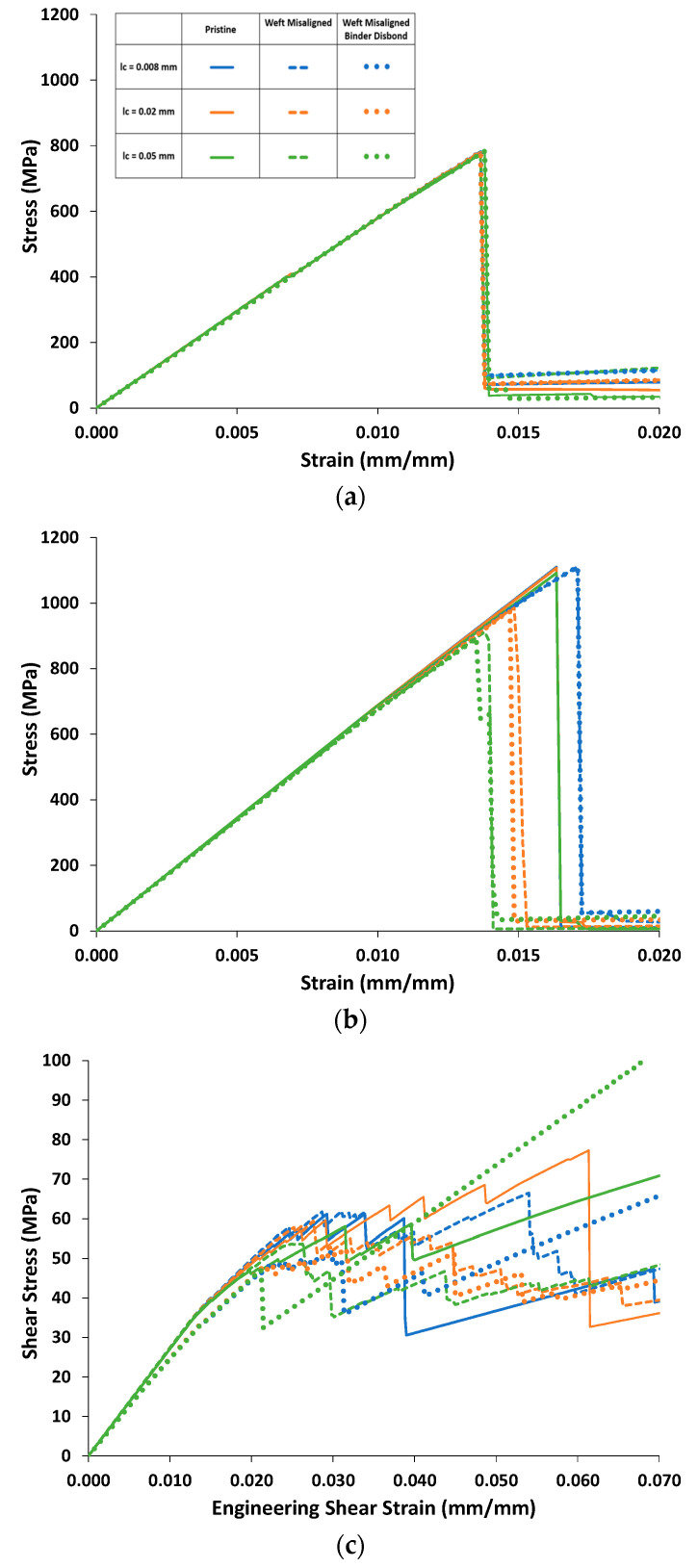
Predicted stress–strain curves for (**a**) warp, (**b**) weft, and (**c**) in-plane shear loading for pristine, weft-misaligned, and weft-misaligned-with-disbond models.

Figure 15 shows predicted stress–strain curves for warp, weft, and in-plane shear loading for the pristine model and models accounting for misaligned weft tows (with and without binder-tow disbonds) with increasing *l_c_*. Predicted effective properties and strengths are shown for these models in Table 9 and Table 10, respectively. For warp-direction loading (Figure 15a), there is little difference between the curves, similar to the results shown in Figure 14a, and only a slight difference in the UTSs (Table 10). However, for the weft-direction loading (Figure 15b), the strength significantly decreased with increasing *l_c_* for models containing misaligned weft tows. For instance, the weft-direction UTS for misaligned weft tows without disbonds decreased from 1111 MPa to 912 MPa as *l_c_* increased from 0.008 to 0.05 (Table 10). Similar values were obtained for the misaligned weft-tow case with disbonds included as well, as shown in Table 10. In these cases, the slight misalignment appeared to make the weft tensile behavior more sensitive to the nonlinear matrix behavior, which was affected significantly by *l_c_*. The trends in the weft stress–strain curves thus followed the trends shown in Figure 13 based on the choice of *l_c_*.

The in-plane shear-response predictions in Figure 15 show that weft-tow misalignment has a relatively minor effect. The in-plane shear stiffness increases slightly (by 1.3%; see Table 9), and the stress–strain curves are slightly higher than the pristine curves just after damage initiation (strains between 0.015 and 0.025). In Table 10, once again, because the shear stress at 5% strain is dependent on the damage-event sequence, the trend in these data is not consistent.

When the slight weft-tow misalignment was added, overall, the effective properties were not significantly different from those in the pristine case (Table 9). However, when disbonds were added, most of the effective properties were reduced, with noticeable decreases in *G*_23_, *G*_13_, and *ν*_23_. This response was consistent with that of the pristine model with and without disbonds (Table 7).

### 4.3. Correlations with Test Data

**Figure 16:**
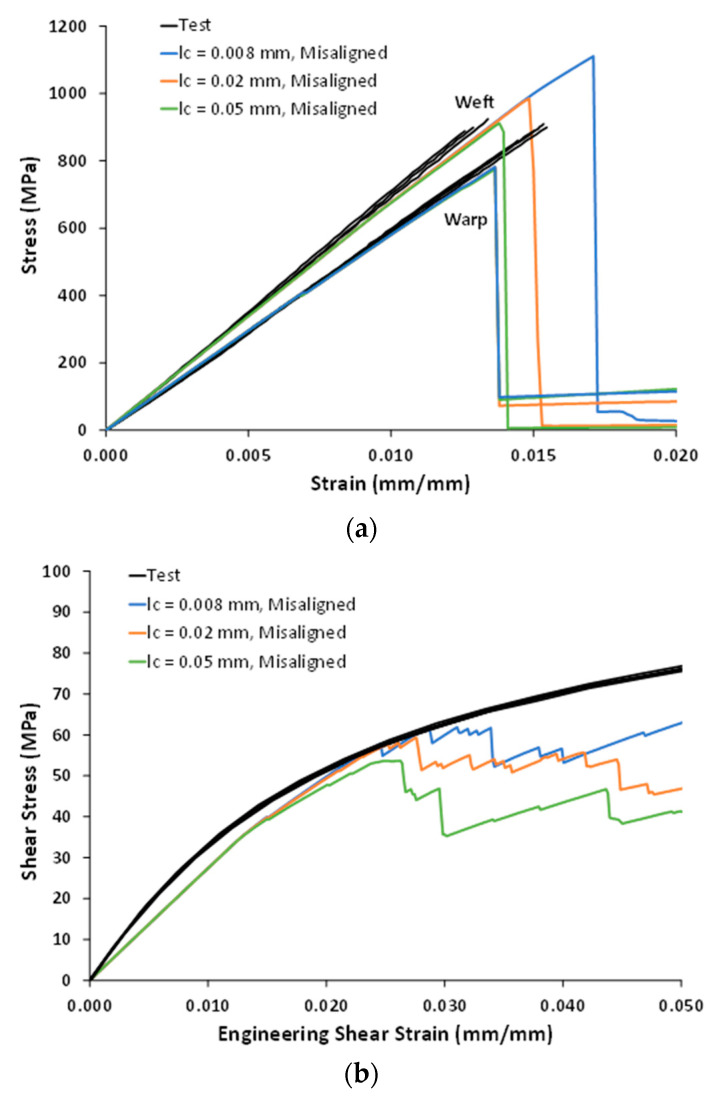
Predicted in-plane nonlinear stress–strain responses of the 3D woven composite for different values of the characteristic length, *l_c_*. (**a**) Warp and weft tension. (**b**) In-plane shear.

Figure 16 compares the in-plane tensile and shear test data with the model results for the misaligned weft tows with no disbonding, including the different values of *l_c_*. The elastic properties and strengths are compared in Table 11 and Table 12. The model did an excellent job of predicting the warp and weft tensile moduli, which were within 5% of the test-data averages. The warp UTS was underpredicted by approximately 13% across all values of *l_c_*. Note that the warp UTS was observed to be insensitive not only to *l_c_* but also to the presence of binder-tow disbonds and weft-tow misalignment. As such, further investigation of both the test and model results is needed to fully understand this discrepancy. In contrast, the weft UTS sensitivity to *l_c_* in the presence of weft-tow misalignment enabled the weft UTS predictions to improve considerably with increasing *l_c_*. With *l_c_* values of 0.02 mm and 0.05 mm, the weft UTS predictions bounded the test data, with both predictions within 7%.

Figure 16b shows that, while the model was able to capture the general, less brittle character of the in-plane shear response, it significantly underpredicted the in-plane shear modulus and shear stress at 5% strain. Regardless of the value of *l_c_*, the in-plane shear stress–strain curves were also underpredicted. It should be noted that the model’s only nonlinear deformation mechanism is stiffness-reduction damage, whereas other nonlinear mechanisms (e.g., plasticity, viscoelasticity) could be present in shear tests. Additionally, model refinement could improve the in-plane shear predictions, particularly if the actual geometry of the weft tows (i.e., their waviness) is accurately captured.

## 5. Conclusions

A multiscale progressive failure model for an IM7/6k carbon fiber–RTM6 epoxy 3D orthogonal woven composite has been presented. Based on the MsRM approach, the methodology employed considered the fiber and matrix constituents within the tows, as well as the intertow matrix. The GMC and HFGMC micromechanical theories were used at different scales, while a crack-band progressive damage model was used for the matrix material. The model RUC dimensions and local fiber volume fractions were determined via measurements taken from X-ray CT and SEM images. Two key features observed in these images, binder-tow disbonding and weft-tow misalignment, were also incorporated into the models.

Progressive damage simulations were conducted for applied warp and weft tensions, as well as applied in-plane shear stresses. Stiffness and strength predictions, along with full stress–strain curves, were compared for different values of the crack-band length-scale parameter, *l_c_*, for cases with and without binder-tow disbonding and with and without weft-tow misalignment. It was found that both the disbonding and the choice of *l_c_* had very minimal effects on the fairly brittle warp and weft tensile behavior. However, the simulated in-plane shear response, which was much more ductile, was quite sensitive to the choice of *l_c_* and the presence of the binder-tow disbonding. The impact of weft-fiber misalignment was small on the warp-direction tensile response and relatively minor on the in-plane shear response, but quite significant on the weft-direction tensile behavior. With the addition of the weft-tow misalignment, a strong influence of the choice of *l_c_* on the simulated weft-direction tensile response was observed, indicating that the misalignment greatly enhanced the influence of the matrix nonlinearity. 

Finally, comparing the predictions with experimental data, it was shown that the model’s stiffness predictions were in very good agreement with the test data for in-plane tension, while the in-plane shear modulus was underpredicted to some extent. Tensile strength predictions were also reasonable, with the weft-tow misalignment enabling much-improved strength predictions for the weft direction. Most notable was the clear ability of the presented multiscale model to capture the fairly brittle response for in-plane tension, while also capturing the much more ductile in-plane shear response observed in the experiments.

## Figures and Tables

**Table 1:** Material properties employed for the IM7 fiber and RTM6 resin within the 3D woven-composite model.

IM7 Carbon Fiber [26]		RTM6 Epoxy Resin		
Property	Value	Property	Value	Source
*E_11f_* (GPa)	262.2	*E* (GPa)	2.755	[40]
*E_22f_* (GPa)	11.8	*ν*	0.38	[40]
*G_12f_* (GPa)	18.9	*X_m_* (MPa)	87	[40]
*ν_12f_*	0.17	*Y_m_* (MPa)	76.1	[40]
*ν_23f_*	0.21	*G*_IC_ (N-mm/mm)	0.216	[19]
*X_11f_* (MPa)	4335	*G*_IIC_ (N-mm/mm)	0.857	[19]
*X_22f_* (MPa)	113			
*Y_11f_* (MPa)	2608			
*Y_22f_* (MPa)	354			
*Z_12f_* (MPa)	138			
*Z_23f_* (MPa)	128			

**Table 2:** Three-dimensional woven-composite RUC geometric dimensions, as measured from X-ray CT images.

Variable	Description	Mean (mm)	Standard Deviation (mm)
WaW	Warp-tow width	1.108	0.068
WaH	Warp-tow height	0.186	0.022
WeW	Weft-tow width	1.186	0.118
WeH	Weft-tow height	0.202	0.026
BWf	Binder-tow width (flat region)	1.171	0.081
BHf	Binder-tow height (flat region)	0.174	0.017
BWz	Binder-tow width (TT region)	0.501	0.105
BHz	Binder-tow height (TT region)	0.413	0.052

**Table 3:** SEM image segmentation results for the fiber volume fraction (V_f_) within the warp and weft tows of the 3D woven composite.

	Number of Measurements	Mean V_f_	V_f_ Standard Deviation
Warp Tows	66	0.6705	0.0249
Weft Tows	54	0.6738	0.0876
Combined	120	0.6720	0.0617

**Table 4:** Warp-direction tensile-test tensile modulus and strength data.

	Strain Gauge Tensile Modulus (GPa)	DIC Tensile Modulus (GPa)	Strength (MPa)
	58.066	57.549	915
	58.497	56.173	892
	59.778	56.537	906
	58.575	57.864	871
	58.960	-	924
Avg.	58.775	57.031	902
St. Dev.	0.644	0.805	21

**Table 5:** Weft-direction tensile-test tensile modulus and strength data.

	Strain Gauge Tensile Modulus (GPa)	DIC Tensile Modulus (GPa)	Strength (MPa)
	67.754	-	970
	67.088	68.137	933
	66.680	-	905
	69.624	70.919	894
	69.198	70.481	909
Avg.	68.069	69.846	922
St. Dev.	1.293	1.496	30

**Table 6:** In-plane shear-test shear modulus and shear stress at 5% strain data.

	Strain Gauge Shear Modulus (GPa)	Shear Stress at 5% Strain (MPa)
	3.369	71.881
	3.425	72.802
	3.271	71.360
	3.326	71.808
Avg.	3.348	71.968
St. Dev.	0.065	0.599

**Table 7:** Predicted effective elastic properties of the 3D woven composite with and without binder-tow disbonds (1—TT; 2—weft; 3—warp).

	Pristine	Disbonded Binder Tow	Difference
*E*_11_ (GPa)	8.51	8.46	−0.6%
*E*_22_ (GPa)	68.9	68.9	0%
*E*_33_ (GPa)	59.2	58.5	−1.2%
*G*_23_ (GPa)	2.71	2.49	−8.1%
*G*_13_ (GPa)	1.88	1.72	−8.5%
*G*_12_ (GPa)	1.95	1.94	−0.5%
*ν*_23_	0.0315	0.0291	−7.6%
*ν*_13_	0.0492	0.0483	−1.8%
*ν*_12_	0.0411	0.0410	−0.2%

**Table 8:** Predicted warp and weft UTSs and in-plane shear stresses at 5% strain as functions of the specified characteristic length, $l_{c}$, with and without binder-tow disbonds.

	*l_c_* (mm)	Warp UTS (MPa)	Weft UTS (MPa)	In-Plane Shear Stress at 5% Strain (MPa)
Pristine	0.008	782	1110	36.7
	0.02	780	1107	65.2
	0.05	781	1092	57.2
Disbonded	0.008	781	1109	48.2
Binder	0.02	788	1107	49.0
Tow	0.05	780	1091	41.9

**Table 9:** Predicted effective elastic properties of the 3D woven composite with and without weft-tow misalignment, in addition to binder-tow disbonds (1—TT; 2—weft; 3—warp).

	Pristine	Weft Misalignment	Difference vs. Pristine	Weft Misalignment and Binder Disbond	Difference vs. Pristine
*E*_11_ (GPa)	8.51	8.52	0.1%	8.46	−0.6%
*E*_22_ (GPa)	68.9	67.9	−1.5%	67.8	−1.6%
*E*_33_ (GPa)	59.2	59.3	0.2%	58.6	−1.0%
*G*_23_ (GPa)	2.71	2.75	1.3%	2.52	−7.0%
*G*_13_ (GPa)	1.88	1.89	0.5%	1.73	−8.0%
*G*_12_ (GPa)	1.95	1.95	0%	1.95	0%
*ν*_23_	0.0315	0.0320	1.6%	0.0296	−6.3%
*ν*_13_	0.0492	0.0491	−0.2%	0.0482	−2.0%
*ν*_12_	0.0411	0.0418	1.7%	0.0416	1.2%

**Table 10:** Predicted warp and weft UTSs and in-plane shear stresses at 5% strain as functions of the specified characteristic length, *l_c_*, with and without tow misalignment, in addition to binder-tow disbonds.

	*l_c_* (mm)	Warp UTS (MPa)	Weft UTS (MPa)	In-Plane Shear Stress at 5% Strain (MPa)
Pristine	0.008	782	1110	36.7
	0.02	780	1107	65.2
	0.05	781	1092	57.2
Misaligned Weft	0.008	782	1111	62.9
Tows	0.02	781	985	46.8
	0.05	775	912	41.3
Misaligned Weft	0.008	782	1111	48.7
Tows and Binder	0.02	781	974	44.0
Disbond	0.05	783	894	73.5

**Table 11:** Comparison between test and predicted composite elastic properties.

	Test Avg.	Model	Difference
Warp Tensile Modulus (GPa)	57.031	59.3	4.0%
Weft Tensile Modulus (GPa)	69.846	67.9	−2.8%
In-Plane Shear Modulus (GPa)	3.348	2.75	−17.9%

**Table 12:** Comparison between test and predicted composite strength data.

	Test Avg.	Model *l_c_* = 0.008 mm	Difference	Model *l_c_* = 0.02 mm	Difference	Model *l_c_* = 0.05 mm	Difference
Warp UTS (MPa)	902	782	−13.3%	781	−13.4%	775	−14.1%
Weft UTS (MPa)	922	1111	20.5%	985	6.8%	912	−1.1%
In-Plane Shear Stress at 5% Strain (MPa)	71.968	62.9	−12.6%	46.8	−35.0%	41.3	−42.6%

## Data Availability

Some data presented in this study are available on request from the corresponding author. Some data are not publicly available due to NASA and U.S. government restrictions.

## Nomenclature

**Variable**	**Description**
$A_{i}^{\left(\alpha_{i}\right)},C_{i}^{\left(\alpha_{i}\right)}$	Strain concentration and stiffness tensors for subvolume *α_i_*, respectively
$C_{i}^{*}$	Effective stiffness tensor for an RUC at Level *i*
BHf, BWf	Binder-tow height and width, flat warp-aligned region, respectively
BHz, BWz	Binder-tow height and width, mid-thickness region, respectively
*D_M_*	Scalar damage variable
*E, G*	Undamaged matrix Young’s and shear moduli, respectively
*E_11f_*, *E_22f_*, *G_12f_*, *ν_12f_*, *ν_23f_*	Transversely isotropic fiber elastic constants
*E*_11_, *E*_22_, *E*_33_	Orthotropic elastic Young’s moduli
*G*_23_, *G*_13_, *G*_12_	Orthotropic elastic shear moduli
*l* *_c_*	Characteristic length
*G_I_, G_II_*	Mode-I- and mode-II-strain energy-release rates, respectively
*G_IC_, G_IIC_*	Mode-I and mode-II fracture toughnesses, respectively
$N_{\alpha_{i}}$	Total number of subvolumes
$S^{\left(\alpha_{m}\right)}$	Damaged compliance matrix
$t_{I}$, $t_{II}$	Mode-I and mode-II crack-band tractions, respectively
*t_M,_ ε_M_*	Mixed-mode traction and strain, respectively
$t_{M}^{0}$ *,* $\varepsilon_{M}^{0}$	Mixed-mode traction and strain when damage initiates, respectively
$T_{2}^{i},T_{4}^{i}$	Second- and fourth-order coordinate transformation tensors for RUC at Level *i*, respectively
$v_{\alpha_{i}}$	Volume fraction of subvolume *α_i_*
WaH, WaW	Warp-tow height and width, respectively
WeH, WeW	Weft-tow height and width, respectively
$X_{m}$, $Y_{m}$	Normal and shear cohesive strength (matrixes), respectively
*α_i_*	Subvolume located at Level *i*
$\varepsilon_{i}^{\left(\alpha_{i}\right)},\sigma_{i}^{\left(\alpha_{i}\right)}$	Subvolume *α_i_* strain and stress tensors, respectively
${\bar{\varepsilon }}_{i},{\bar{\sigma }}_{i}$	Average strain and stress tensors for an RUC at Level *i*, respectively
$\varepsilon_{I\;},\;\gamma_{II}$	Mode-I and mode-II crack-band strains, respectively
$\varepsilon_{M}^{0}$, $\varepsilon_{M}^{f}$	Mixed-mode damage initiation and failure strains, respectively
$\sigma_{ii}^{\left(\alpha_{m}\right)},\tau_{ij}^{\left(\alpha_{m}\right)}$	*ii* normal and *ij* shear stresses for matrix subvolume $\alpha_{m}$, respectively
*ν*	Poisson ratio for isotropic matrix
*ν*_23_,*ν*_23_,*ν*_12_	Orthotropic elastic Poisson ratios
